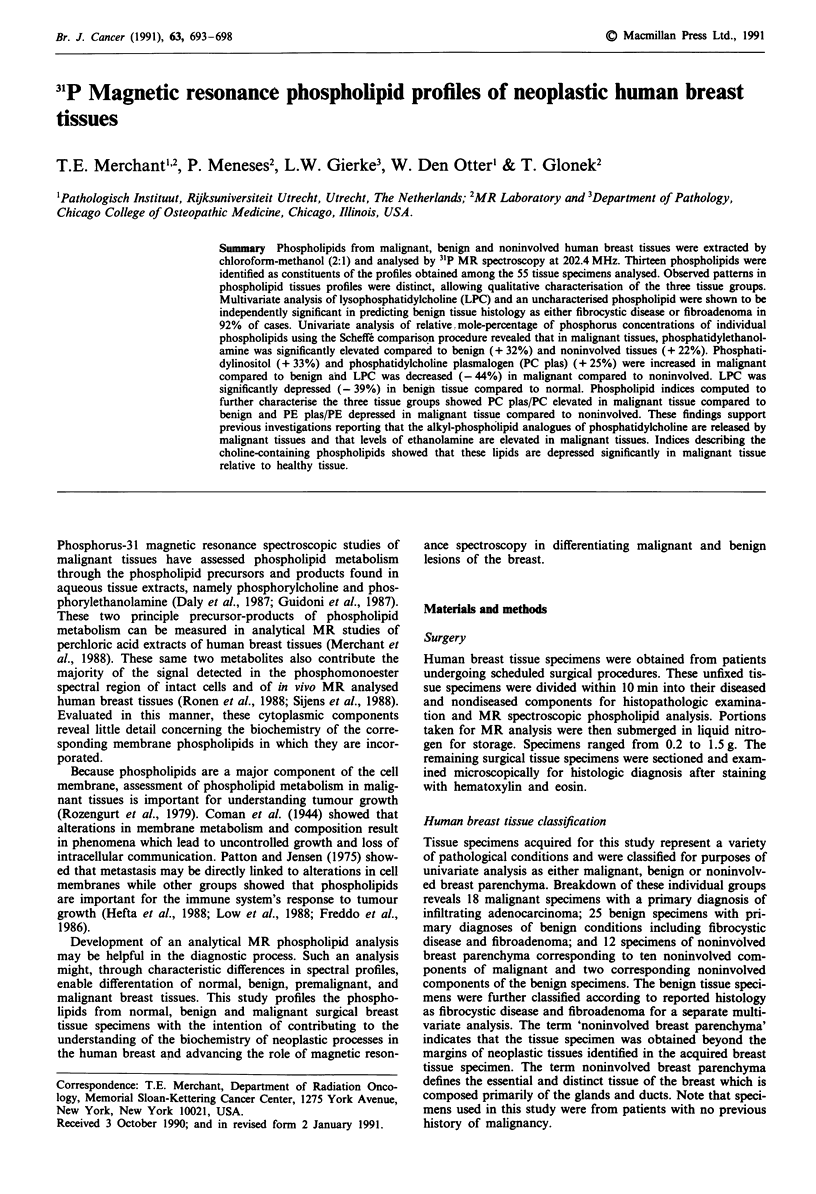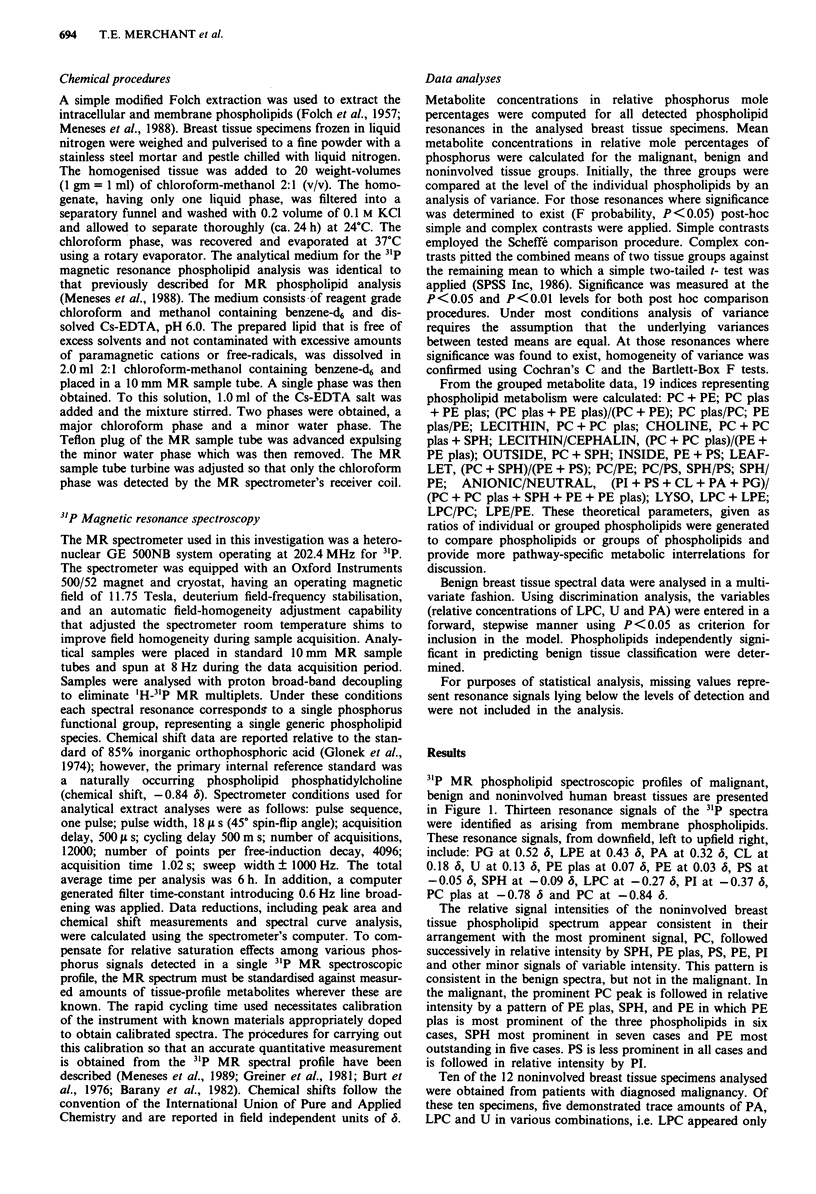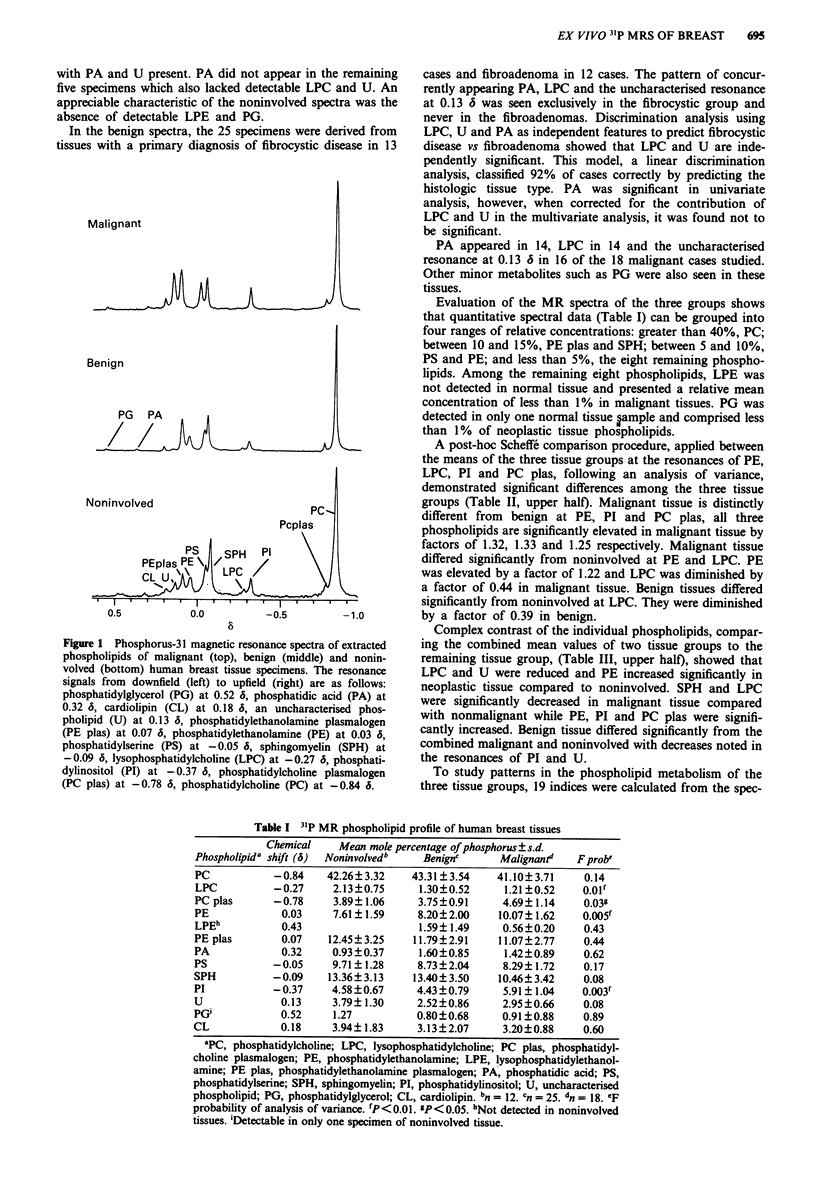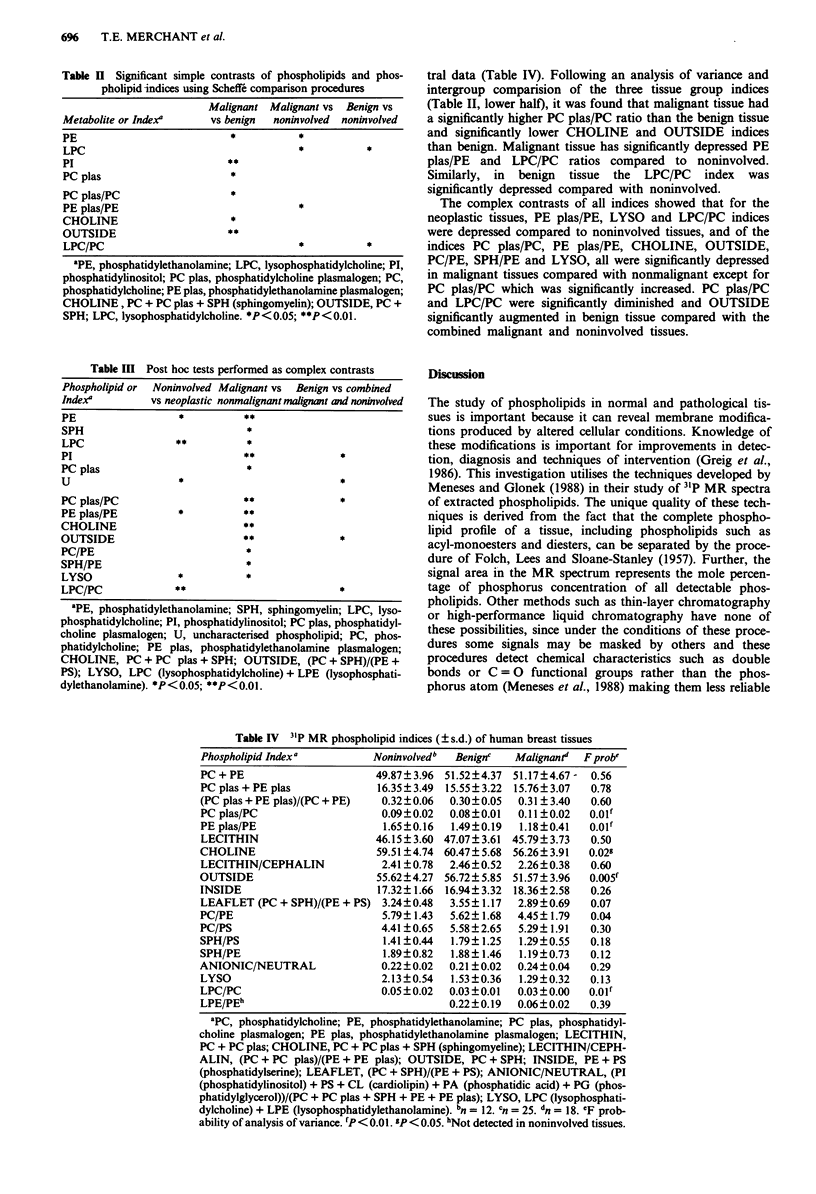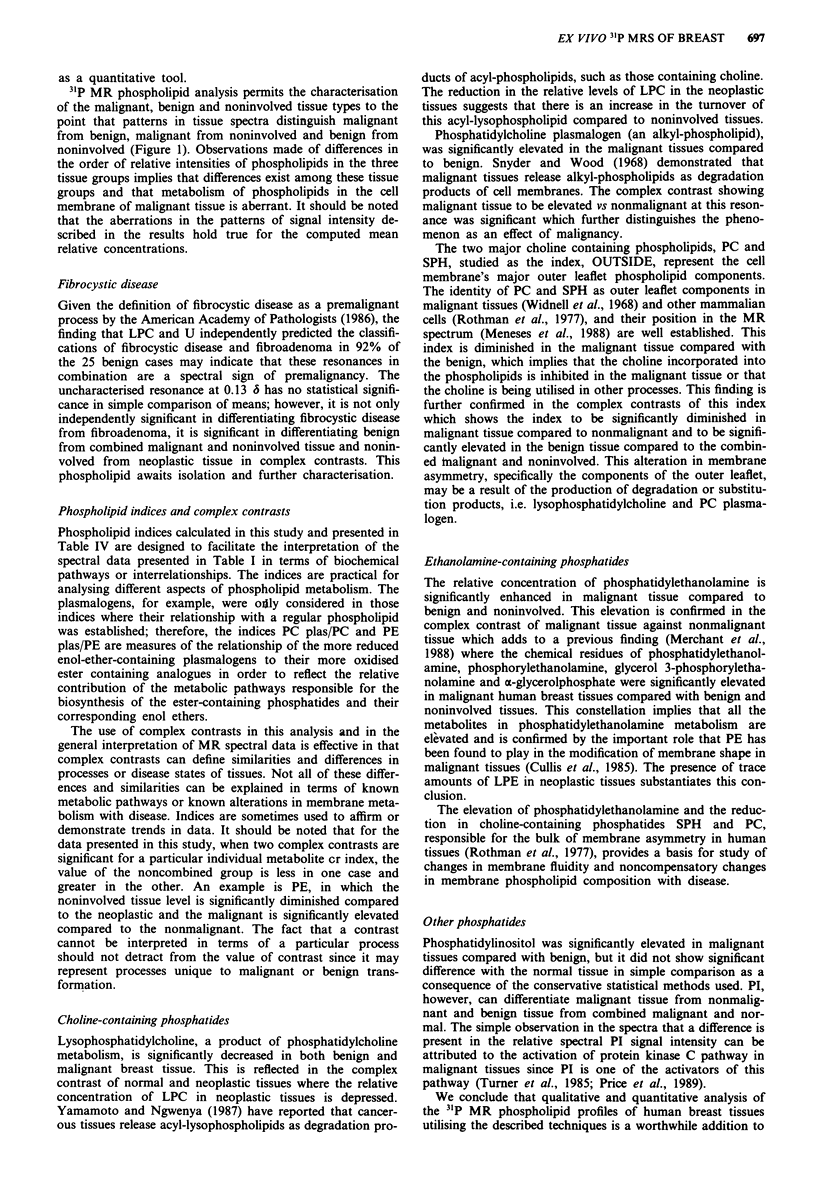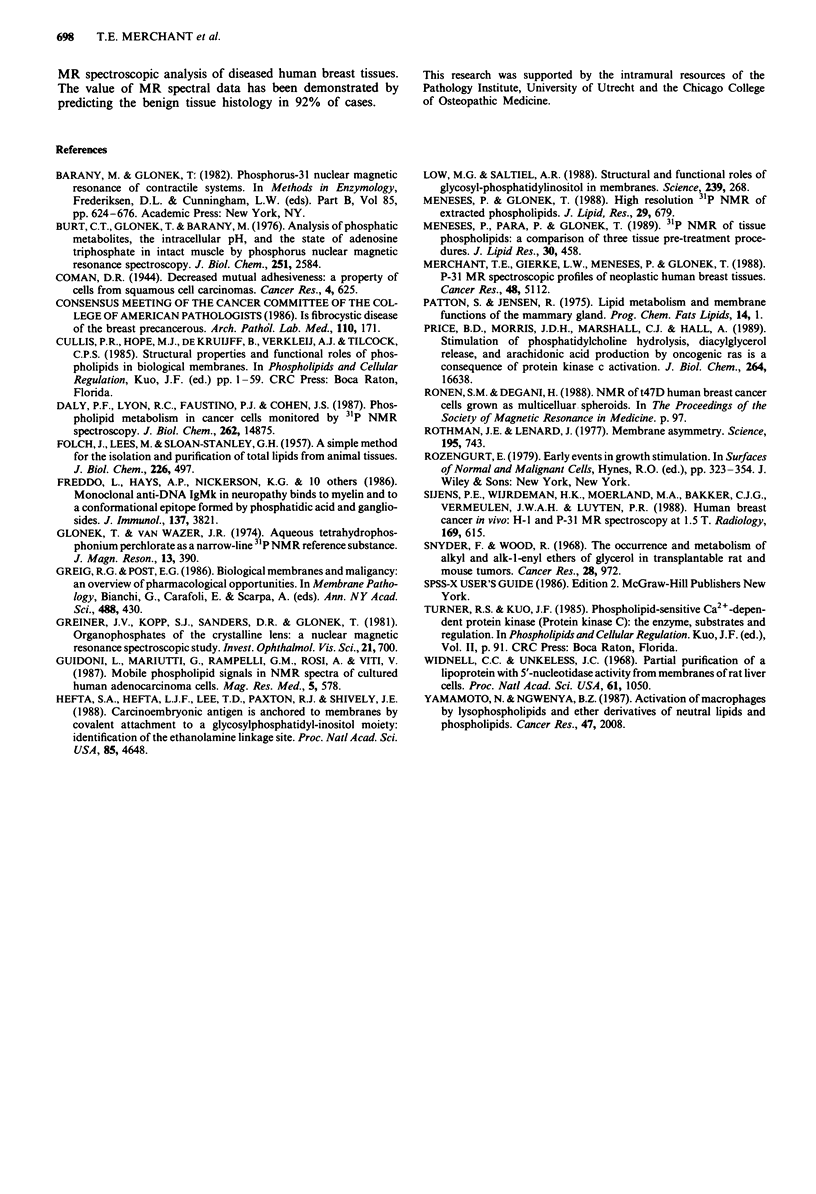# 31P magnetic resonance phospholipid profiles of neoplastic human breast tissues.

**DOI:** 10.1038/bjc.1991.157

**Published:** 1991-05

**Authors:** T. E. Merchant, P. Meneses, L. W. Gierke, W. Den Otter, T. Glonek

**Affiliations:** Pathologisch Instituut, Rijksuniversiteit Utrecht, The Netherlands.

## Abstract

Phospholipids from malignant, benign and noninvolved human breast tissues were extracted by chloroform-methanol (2:1) and analysed by 31P MR spectroscopy at 202.4 MHz. Thirteen phospholipids were identified as constituents of the profiles obtained among the 55 tissue specimens analysed. Observed patterns in phospholipid tissues profiles were distinct, allowing qualitative characterisation of the three tissue groups. Multivariate analysis of lysophosphatidylcholine (LPC) and an uncharacterised phospholipid were shown to be independently significant in predicting benign tissue histology as either fibrocystic disease or fibroadenoma in 92% of cases. Univariate analysis of relative mole-percentage of phosphorus concentrations of individual phospholipids using the Scheffé comparison procedure revealed that in malignant tissues, phosphatidylethanolamine was significantly elevated compared to benign (+ 32%) and noninvolved tissues (+ 22%). Phosphatidylinositol (+ 33%) and phosphatidylcholine plasmalogen (PC plas) (+ 25%) were increased in malignant compared to benign and LPC was decreased (-44%) in malignant compared to noninvolved. LPC was significantly depressed (-39%) in benign tissue compared to normal. Phospholipid indices computed to further characterise the three tissue groups showed PC plas/PC elevated in malignant tissue compared to benign and PE plas/PE depressed in malignant tissue compared to noninvolved. These findings support previous investigations reporting that the alkyl-phospholipid analogues of phosphatidylcholine are released by malignant tissues and that levels of ethanolamine are elevated in malignant tissues. Indices describing the choline-containing phospholipids showed that these lipids are depressed significantly in malignant tissue relative to healthy tissue.


Br. J. Cancer (1991), 63, 693-698 © Macmillan Press Ltd., 1991

### $^{31}$P Magnetic resonance phospholipid profiles of neoplastic human breast tissues

T.E. Merchant[1,2], P. Meneses[2], L.W. Gierke[3], W. Den Otter[1] & T. Glonek[2]

[1]*Pathologisch Instituut, Rijksuniversiteit Utrecht, Utrecht, The Netherlands;* [2]*MR Laboratory and* [3]*Department of Pathology, Chicago College of Osteopathic Medicine, Chicago, Illinois, USA.*

**Summary** Phospholipids from malignant, benign and noninvolved human breast tissues were extracted by chloroform-methanol (2:1) and analysed by $^{31}$P MR spectroscopy at 202.4 MHz. Thirteen phospholipids were identified as constituents of the profiles obtained among the 55 tissue specimens analysed. Observed patterns in phospholipid tissues profiles were distinct, allowing qualitative characterisation of the three tissue groups. Multivariate analysis of lysophosphatidylcholine (LPC) and an uncharacterised phospholipid were shown to be independently significant in predicting benign tissue histology as either fibrocystic disease or fibroadenoma in 92% of cases. Univariate analysis of relative mole-percentage of phosphorus concentrations of individual phospholipids using the Scheffé comparison procedure revealed that in malignant tissues, phosphatidylethanolamine was significantly elevated compared to benign (+ 32%) and noninvolved tissues (+ 22%). Phosphatidylinositol (+ 33%) and phosphatidylcholine plasmalogen (PC plas) (+ 25%) were increased in malignant compared to benign and LPC was decreased (– 44%) in malignant compared to noninvolved. LPC was significantly depressed (– 39%) in benign tissue compared to normal. Phospholipid indices computed to further characterise the three tissue groups showed PC plas/PC elevated in malignant tissue compared to benign and PE plas/PE depressed in malignant tissue compared to noninvolved. These findings support previous investigations reporting that the alkyl-phospholipid analogues of phosphatidylcholine are released by malignant tissues and that levels of ethanolamine are elevated in malignant tissues. Indices describing the choline-containing phospholipids showed that these lipids are depressed significantly in malignant tissue relative to healthy tissue.

Phosphorus-31 magnetic resonance spectroscopic studies of malignant tissues have assessed phospholipid metabolism through the phospholipid precursors and products found in aqueous tissue extracts, namely phosphorylcholine and phosphorylethanolamine (Daly *et al.*, 1987; Guidoni *et al.*, 1987). These two principle precursor-products of phospholipid metabolism can be measured in analytical MR studies of perchloric acid extracts of human breast tissues (Merchant *et al.*, 1988). These same two metabolites also contribute the majority of the signal detected in the phosphomonoester spectral region of intact cells and of *in vivo* MR analysed human breast tissues (Ronen *et al.*, 1988; Sijens *et al.*, 1988). Evaluated in this manner, these cytoplasmic components reveal little detail concerning the biochemistry of the corresponding membrane phospholipids in which they are incorporated.

Because phospholipids are a major component of the cell membrane, assessment of phospholipid metabolism in malignant tissues is important for understanding tumour growth (Rozengurt *et al.*, 1979). Coman *et al.* (1944) showed that alterations in membrane metabolism and composition result in phenomena which lead to uncontrolled growth and loss of intracellular communication. Patton and Jensen (1975) showed that metastasis may be directly linked to alterations in cell membranes while other groups showed that phospholipids are important for the immune system's response to tumour growth (Hefta *et al.*, 1988; Low *et al.*, 1988; Freddo *et al.*, 1986).

Development of an analytical MR phospholipid analysis may be helpful in the diagnostic process. Such an analysis might, through characteristic differences in spectral profiles, enable differentation of normal, benign, premalignant, and malignant breast tissues. This study profiles the phospholipids from normal, benign and malignant surgical breast tissue specimens with the intention of contributing to the understanding of the biochemistry of neoplastic processes in the human breast and advancing the role of magnetic resonance spectroscopy in differentiating malignant and benign lesions of the breast.

Correspondence: T.E. Merchant, Department of Radiation Oncology, Memorial Sloan-Kettering Cancer Center, 1275 York Avenue, New York, New York 10021, USA.
Received 3 October 1990; and in revised form 2 January 1991.

### Materials and methods

### *Surgery*

Human breast tissue specimens were obtained from patients undergoing scheduled surgical procedures. These unfixed tissue specimens were divided within 10 min into their diseased and nondiseased components for histopathologic examination and MR spectroscopic phospholipid analysis. Portions taken for MR analysis were then submerged in liquid nitrogen for storage. Specimens ranged from 0.2 to 1.5 g. The remaining surgical tissue specimens were sectioned and examined microscopically for histologic diagnosis after staining with hematoxylin and eosin.

### *Human breast tissue classification*

Tissue specimens acquired for this study represent a variety of pathological conditions and were classified for purposes of univariate analysis as either malignant, benign or noninvolved breast parenchyma. Breakdown of these individual groups reveals 18 malignant specimens with a primary diagnosis of infiltrating adenocarcinoma; 25 benign specimens with primary diagnoses of benign conditions including fibrocystic disease and fibroadenoma; and 12 specimens of noninvolved breast parenchyma corresponding to ten noninvolved components of malignant and two corresponding noninvolved components of the benign specimens. The benign tissue specimens were further classified according to reported histology as fibrocystic disease and fibroadenoma for a separate multivariate analysis. The term 'noninvolved breast parenchyma' indicates that the tissue specimen was obtained beyond the margins of neoplastic tissues identified in the acquired breast tissue specimen. The term noninvolved breast parenchyma defines the essential and distinct tissue of the breast which is composed primarily of the glands and ducts. Note that specimens used in this study were from patients with no previous history of malignancy.

*Chemical procedures*

A simple modified Folch extraction was used to extract the intracellular and membrane phospholipids (Folch et al., 1957; Meneses et al., 1988). Breast tissue specimens frozen in liquid nitrogen were weighed and pulverised to a fine powder with a stainless steel mortar and pestle chilled with liquid nitrogen. The homogenised tissue was added to 20 weight-volumes (1 gm = 1 ml) of chloroform-methanol 2:1 (v/v). The homogenate, having only one liquid phase, was filtered into a separatory funnel and washed with 0.2 volume of 0.1 M KCl and allowed to separate thoroughly (ca. 24 h) at 24°C. The chloroform phase, was recovered and evaporated at 37°C using a rotary evaporator. The analytical medium for the $^{31}P$ magnetic resonance phospholipid analysis was identical to that previously described for MR phospholipid analysis (Meneses et al., 1988). The medium consists of reagent grade chloroform and methanol containing benzene-$d_6$ and dissolved Cs-EDTA, pH 6.0. The prepared lipid that is free of excess solvents and not contaminated with excessive amounts of paramagnetic cations or free-radicals, was dissolved in 2.0 ml 2:1 chloroform-methanol containing benzene-$d_6$ and placed in a 10 mm MR sample tube. A single phase was then obtained. To this solution, 1.0 ml of the Cs-EDTA salt was added and the mixture stirred. Two phases were obtained, a major chloroform phase and a minor water phase. The Teflon plug of the MR sample tube was advanced expulsing the minor water phase which was then removed. The MR sample tube turbine was adjusted so that only the chloroform phase was detected by the MR spectrometer's receiver coil.

*$^{31}P$ Magnetic resonance spectroscopy*

The MR spectrometer used in this investigation was a heteronuclear GE 500NB system operating at 202.4 MHz for $^{31}P$. The spectrometer was equipped with an Oxford Instruments 500/52 magnet and cryostat, having an operating magnetic field of 11.75 Tesla, deuterium field-frequency stabilisation, and an automatic field-homogeneity adjustment capability that adjusted the spectrometer room temperature shims to improve field homogeneity during sample acquisition. Analytical samples were placed in standard 10 mm MR sample tubes and spun at 8 Hz during the data acquisition period. Samples were analysed with proton broad-band decoupling to eliminate $^{1}H$-$^{31}P$ MR multiplets. Under these conditions each spectral resonance corresponds to a single phosphorus functional group, representing a single generic phospholipid species. Chemical shift data are reported relative to the standard of 85% inorganic orthophosphoric acid (Glonek et al., 1974); however, the primary internal reference standard was a naturally occurring phospholipid phosphatidylcholine (chemical shift, $-0.84\ \delta$). Spectrometer conditions used for analytical extract analyses were as follows: pulse sequence, one pulse; pulse width, 18 $\mu$ s (45° spin-flip angle); acquisition delay, 500 $\mu$ s; cycling delay 500 m s; number of acquisitions, 12000; number of points per free-induction decay, 4096; acquisition time 1.02 s; sweep width $\pm$ 1000 Hz. The total average time per analysis was 6 h. In addition, a computer generated filter time-constant introducing 0.6 Hz line broadening was applied. Data reductions, including peak area and chemical shift measurements and spectral curve analysis, were calculated using the spectrometer's computer. To compensate for relative saturation effects among various phosphorus signals detected in a single $^{31}P$ MR spectroscopic profile, the MR spectrum must be standardised against measured amounts of tissue-profile metabolites wherever these are known. The rapid cycling time used necessitates calibration of the instrument with known materials appropriately doped to obtain calibrated spectra. The procedures for carrying out this calibration so that an accurate quantitative measurement is obtained from the $^{31}P$ MR spectral profile have been described (Meneses et al., 1989; Greiner et al., 1981; Burt et al., 1976; Barany et al., 1982). Chemical shifts follow the convention of the International Union of Pure and Applied Chemistry and are reported in field independent units of $\delta$.

*Data analyses*

Metabolite concentrations in relative phosphorus mole percentages were computed for all detected phospholipid resonances in the analysed breast tissue specimens. Mean metabolite concentrations in relative mole percentages of phosphorus were calculated for the malignant, benign and noninvolved tissue groups. Initially, the three groups were compared at the level of the individual phospholipids by an analysis of variance. For those resonances where significance was determined to exist (F probability, $P<0.05$) post-hoc simple and complex contrasts were applied. Simple contrasts employed the Scheffé comparison procedure. Complex contrasts pitted the combined means of two tissue groups against the remaining mean to which a simple two-tailed $t$- test was applied (SPSS Inc, 1986). Significance was measured at the $P<0.05$ and $P<0.01$ levels for both post hoc comparison procedures. Under most conditions analysis of variance requires the assumption that the underlying variances between tested means are equal. At those resonances where significance was found to exist, homogeneity of variance was confirmed using Cochran's C and the Bartlett-Box F tests.

From the grouped metabolite data, 19 indices representing phospholipid metabolism were calculated: PC + PE; PC plas + PE plas; (PC plas + PE plas)/(PC + PE); PC plas/PC; PE plas/PE; LECITHIN, PC + PC plas; CHOLINE, PC + PC plas + SPH; LECITHIN/CEPHALIN, (PC + PC plas)/(PE + PE plas); OUTSIDE, PC + SPH; INSIDE, PE + PS; LEAFLET, (PC + SPH)/(PE + PS); PC/PE; PC/PS, SPH/PS; SPH/PE; ANIONIC/NEUTRAL, (PI + PS + CL + PA + PG)/(PC + PC plas + SPH + PE + PE plas); LYSO, LPC + LPE; LPC/PC; LPE/PE. These theoretical parameters, given as ratios of individual or grouped phospholipids were generated to compare phospholipids or groups of phospholipids and provide more pathway-specific metabolic interrelations for discussion.

Benign breast tissue spectral data were analysed in a multivariate fashion. Using discrimination analysis, the variables (relative concentrations of LPC, U and PA) were entered in a forward, stepwise manner using $P<0.05$ as criterion for inclusion in the model. Phospholipids independently significant in predicting benign tissue classification were determined.

For purposes of statistical analysis, missing values represent resonance signals lying below the levels of detection and were not included in the analysis.

### Results

$^{31}P$ MR phospholipid spectroscopic profiles of malignant, benign and noninvolved human breast tissues are presented in Figure 1. Thirteen resonance signals of the $^{31}P$ spectra were identified as arising from membrane phospholipids. These resonance signals, from downfield, left to upfield right, include: PG at 0.52 $\delta$, LPE at 0.43 $\delta$, PA at 0.32 $\delta$, CL at 0.18 $\delta$, U at 0.13 $\delta$, PE plas at 0.07 $\delta$, PE at 0.03 $\delta$, PS at $-0.05\ \delta$, SPH at $-0.09\ \delta$, LPC at $-0.27\ \delta$, PI at $-0.37\ \delta$, PC plas at $-0.78\ \delta$ and PC at $-0.84\ \delta$.

The relative signal intensities of the noninvolved breast tissue phospholipid spectrum appear consistent in their arrangement with the most prominent signal, PC, followed successively in relative intensity by SPH, PE plas, PS, PE, PI and other minor signals of variable intensity. This pattern is consistent in the benign spectra, but not in the malignant. In the malignant, the prominent PC peak is followed in relative intensity by a pattern of PE plas, SPH, and PE in which PE plas is most prominent of the three phospholipids in six cases, SPH most prominent in seven cases and PE most outstanding in five cases. PS is less prominent in all cases and is followed in relative intensity by PI.

Ten of the 12 noninvolved breast tissue specimens analysed were obtained from patients with diagnosed malignancy. Of these ten specimens, five demonstrated trace amounts of PA, LPC and U in various combinations, i.e. LPC appeared only with PA and U present. PA did not appear in the remaining five specimens which also lacked detectable LPC and U. An appreciable characteristic of the noninvolved spectra was the absence of detectable LPE and PG.

In the benign spectra, the 25 specimens were derived from tissues with a primary diagnosis of fibrocystic disease in 13 cases and fibroadenoma in 12 cases. The pattern of concurrently appearing PA, LPC and the uncharacterised resonance at 0.13 $\delta$ was seen exclusively in the fibrocystic group and never in the fibroadenomas. Discrimination analysis using LPC, U and PA as independent features to predict fibrocystic disease *vs* fibroadenoma showed that LPC and U are independently significant. This model, a linear discrimination analysis, classified 92% of cases correctly by predicting the histologic tissue type. PA was significant in univariate analysis, however, when corrected for the contribution of LPC and U in the multivariate analysis, it was found not to be significant.

PA appeared in 14, LPC in 14 and the uncharacterised resonance at 0.13 $\delta$ in 16 of the 18 malignant cases studied. Other minor metabolites such as PG were also seen in these tissues.

Evaluation of the MR spectra of the three groups shows that quantitative spectral data (Table I) can be grouped into four ranges of relative concentrations: greater than 40%, PC; between 10 and 15%, PE plas and SPH; between 5 and 10%, PS and PE; and less than 5%, the eight remaining phospholipids. Among the remaining eight phospholipids, LPE was not detected in normal tissue and presented a relative mean concentration of less than 1% in malignant tissues. PG was detected in only one normal tissue sample and comprised less than 1% of neoplastic tissue phospholipids.

A post-hoc Scheffé comparison procedure, applied between the means of the three tissue groups at the resonances of PE, LPC, PI and PC plas, following an analysis of variance, demonstrated significant differences among the three tissue groups (Table II, upper half). Malignant tissue is distinctly different from benign at PE, PI and PC plas, all three phospholipids are significantly elevated in malignant tissue by factors of 1.32, 1.33 and 1.25 respectively. Malignant tissue differed significantly from noninvolved at PE and LPC. PE was elevated by a factor of 1.22 and LPC was diminished by a factor of 0.44 in malignant tissue. Benign tissues differed significantly from noninvolved at LPC. They were diminished by a factor of 0.39 in benign.

Complex contrast of the individual phospholipids, comparing the combined mean values of two tissue groups to the remaining tissue group, (Table III, upper half), showed that LPC and U were reduced and PE increased significantly in neoplastic tissue compared to noninvolved. SPH and LPC were significantly decreased in malignant tissue compared with nonmalignant while PE, PI and PC plas were significantly increased. Benign tissue differed significantly from the combined malignant and noninvolved with decreases noted in the resonances of PI and U.

To study patterns in the phospholipid metabolism of the three tissue groups, 19 indices were calculated from the spec-

**Figure 1** Phosphorus-31 magnetic resonance spectra of extracted phospholipids of malignant (top), benign (middle) and noninvolved (bottom) human breast tissue specimens. The resonance signals from downfield (left) to upfield (right) are as follows: phosphatidylglycerol (PG) at 0.52 $\delta$, phosphatidic acid (PA) at 0.32 $\delta$, cardiolipin (CL) at 0.18 $\delta$, an uncharacterised phospholipid (U) at 0.13 $\delta$, phosphatidylethanolamine plasmalogen (PE plas) at 0.07 $\delta$, phosphatidylethanolamine (PE) at 0.03 $\delta$, phosphatidylserine (PS) at $-0.05$ $\delta$, sphingomyelin (SPH) at $-0.09$ $\delta$, lysophosphatidylcholine (LPC) at $-0.27$ $\delta$, phosphatidylinositol (PI) at $-0.37$ $\delta$, phosphatidylcholine plasmalogen (PC plas) at $-0.78$ $\delta$, phosphatidylcholine (PC) at $-0.84$ $\delta$.

**Table I** $^{31}$P MR phospholipid profile of human breast tissues

| Phospholipid[a] | Chemical shift ($\delta$) | Mean mole percentage of phosphorus ± s.d. Noninvolved[b] | Benign[c] | Malignant[d] | F prob[e] |
|---|---|---|---|---|---|
| PC | −0.84 | 42.26±3.32 | 43.31±3.54 | 41.10±3.71 | 0.14 |
| LPC | −0.27 | 2.13±0.75 | 1.30±0.52 | 1.21±0.52 | 0.01[f] |
| PC plas | −0.78 | 3.89±1.06 | 3.75±0.91 | 4.69±1.14 | 0.03[g] |
| PE | 0.03 | 7.61±1.59 | 8.20±2.00 | 10.07±1.62 | 0.005[f] |
| LPE[h] | 0.43 | | 1.59±1.49 | 0.56±0.20 | 0.43 |
| PE plas | 0.07 | 12.45±3.25 | 11.79±2.91 | 11.07±2.77 | 0.44 |
| PA | 0.32 | 0.93±0.37 | 1.60±0.85 | 1.42±0.89 | 0.62 |
| PS | −0.05 | 9.71±1.28 | 8.73±2.04 | 8.29±1.72 | 0.17 |
| SPH | −0.09 | 13.36±3.13 | 13.40±3.50 | 10.46±3.42 | 0.08 |
| PI | −0.37 | 4.58±0.67 | 4.43±0.79 | 5.91±1.04 | 0.003[f] |
| U | 0.13 | 3.79±1.30 | 2.52±0.86 | 2.95±0.66 | 0.08 |
| PG[i] | 0.52 | 1.27 | 0.80±0.68 | 0.91±0.88 | 0.89 |
| CL | 0.18 | 3.94±1.83 | 3.13±2.07 | 3.20±0.88 | 0.60 |

[a]PC, phosphatidylcholine; LPC, lysophosphatidylcholine; PC plas, phosphatidylcholine plasmalogen; PE, phosphatidylethanolamine; LPE, lysophosphatidylethanolamine; PE plas, phosphatidylethanolamine plasmalogen; PA, phosphatidic acid; PS, phosphatidylserine; SPH, sphingomyelin; PI, phosphatidylinositol; U, uncharacterised phospholipid; PG, phosphatidylglycerol; CL, cardiolipin. [b]$n = 12$. [c]$n = 25$. [d]$n = 18$. [e]F probability of analysis of variance. [f]$P < 0.01$. [g]$P < 0.05$. [h]Not detected in noninvolved tissues. [i]Detectable in only one specimen of noninvolved tissue.

**Table II** Significant simple contrasts of phospholipids and phospholipid indices using Scheffé comparison procedures

| *Metabolite or Index*[a] | *Malignant* vs *benign* | *Malignant* vs *noninvolved* | *Benign* vs *noninvolved* |
|---|---|---|---|
| PE | * | * | |
| LPC | | * | * |
| PI | ** | | |
| PC plas | * | | |
| PC plas/PC | * | | |
| PE plas/PE | | * | |
| CHOLINE | * | | |
| OUTSIDE | ** | | |
| LPC/PC | | * | * |

[a]PE, phosphatidylethanolamine; LPC, lysophosphatidylcholine; PI, phosphatidylinositol; PC plas, phosphatidylcholine plasmalogen; PC, phosphatidylcholine; PE plas, phosphatidylethanolamine plasmalogen; CHOLINE , PC + PC plas + SPH (sphingomyelin); OUTSIDE, PC + SPH; LPC, lysophosphatidylcholine. *$P<0.05$; **$P<0.01$.

**Table III** Post hoc tests performed as complex contrasts

| *Phospholipid or Index*[a] | *Noninvolved* vs *neoplastic* | *Malignant* vs *nonmalignant* | *Benign* vs *combined malignant and noninvolved* |
|---|---|---|---|
| PE | * | ** | |
| SPH | | * | |
| LPC | ** | * | |
| PI | | ** | * |
| PC plas | | * | |
| U | * | | * |
| PC plas/PC | | ** | * |
| PE plas/PE | * | ** | |
| CHOLINE | | ** | |
| OUTSIDE | | ** | * |
| PC/PE | | * | |
| SPH/PE | | * | |
| LYSO | * | * | |
| LPC/PC | ** | | * |

[a]PE, phosphatidylethanolamine; SPH, sphingomyelin; LPC, lysophosphatidylcholine; PI, phosphatidylinositol; PC plas, phosphatidylcholine plasmalogen; U, uncharacterised phospholipid; PC, phosphatidylcholine; PE plas, phosphatidylethanolamine plasmalogen; CHOLINE, PC + PC plas + SPH; OUTSIDE, (PC + SPH)/(PE + PS); LYSO, LPC (lysophosphatidylcholine) + LPE (lysophosphatidylethanolamine). *$P<0.05$; **$P<0.01$.

tral data (Table IV). Following an analysis of variance and intergroup comparision of the three tissue group indices (Table II, lower half), it was found that malignant tissue had a significantly higher PC plas/PC ratio than the benign tissue and significantly lower CHOLINE and OUTSIDE indices than benign. Malignant tissue has significantly depressed PE plas/PE and LPC/PC ratios compared to noninvolved. Similarly, in benign tissue the LPC/PC index was significantly depressed compared with noninvolved.

The complex contrasts of all indices showed that for the neoplastic tissues, PE plas/PE, LYSO and LPC/PC indices were depressed compared to noninvolved tissues, and of the indices PC plas/PC, PE plas/PE, CHOLINE, OUTSIDE, PC/PE, SPH/PE and LYSO, all were significantly depressed in malignant tissues compared with nonmalignant except for PC plas/PC which was significantly increased. PC plas/PC and LPC/PC were significantly diminished and OUTSIDE significantly augmented in benign tissue compared with the combined malignant and noninvolved tissues.

### Discussion

The study of phospholipids in normal and pathological tissues is important because it can reveal membrane modifications produced by altered cellular conditions. Knowledge of these modifications is important for improvements in detection, diagnosis and techniques of intervention (Greig *et al.*, 1986). This investigation utilises the techniques developed by Meneses and Glonek (1988) in their study of $^{31}$P MR spectra of extracted phospholipids. The unique quality of these techniques is derived from the fact that the complete phospholipid profile of a tissue, including phospholipids such as acyl-monoesters and diesters, can be separated by the procedure of Folch, Lees and Sloane-Stanley (1957). Further, the signal area in the MR spectrum represents the mole percentage of phosphorus concentration of all detectable phospholipids. Other methods such as thin-layer chromatography or high-performance liquid chromatography have none of these possibilities, since under the conditions of these procedures some signals may be masked by others and these procedures detect chemical characteristics such as double bonds or C = O functional groups rather than the phosphorus atom (Meneses *et al.*, 1988) making them less reliable

**Table IV** $^{31}$P MR phospholipid indices (± s.d.) of human breast tissues

| *Phospholipid Index*[a] | *Noninvolved*[b] | *Benign*[c] | *Malignant*[d] | *F prob*[e] |
|---|---|---|---|---|
| PC + PE | 49.87±3.96 | 51.52±4.37 | 51.17±4.67 | 0.56 |
| PC plas + PE plas | 16.35±3.49 | 15.55±3.22 | 15.76±3.07 | 0.78 |
| (PC plas + PE plas)/(PC + PE) | 0.32±0.06 | 0.30±0.05 | 0.31±3.40 | 0.60 |
| PC plas/PC | 0.09±0.02 | 0.08±0.01 | 0.11±0.02 | 0.01[f] |
| PE plas/PE | 1.65±0.16 | 1.49±0.19 | 1.18±0.41 | 0.01[f] |
| LECITHIN | 46.15±3.60 | 47.07±3.61 | 45.79±3.73 | 0.50 |
| CHOLINE | 59.51±4.74 | 60.47±5.68 | 56.26±3.91 | 0.02[g] |
| LECITHIN/CEPHALIN | 2.41±0.78 | 2.46±0.52 | 2.26±0.38 | 0.60 |
| OUTSIDE | 55.62±4.27 | 56.72±5.85 | 51.57±3.96 | 0.005[f] |
| INSIDE | 17.32±1.66 | 16.94±3.32 | 18.36±2.58 | 0.26 |
| LEAFLET (PC + SPH)/(PE + PS) | 3.24±0.48 | 3.55±1.17 | 2.89±0.69 | 0.07 |
| PC/PE | 5.79±1.43 | 5.62±1.68 | 4.45±1.79 | 0.04 |
| PC/PS | 4.41±0.65 | 5.58±2.65 | 5.29±1.91 | 0.30 |
| SPH/PS | 1.41±0.44 | 1.79±1.25 | 1.29±0.55 | 0.18 |
| SPH/PE | 1.89±0.82 | 1.88±1.46 | 1.19±0.73 | 0.12 |
| ANIONIC/NEUTRAL | 0.22±0.02 | 0.21±0.02 | 0.24±0.04 | 0.29 |
| LYSO | 2.13±0.54 | 1.53±0.36 | 1.29±0.32 | 0.13 |
| LPC/PC | 0.05±0.02 | 0.03±0.01 | 0.03±0.00 | 0.01[f] |
| LPE/PE[h] | | 0.22±0.19 | 0.06±0.02 | 0.39 |

[a]PC, phosphatidylcholine; PE, phosphatidylethanolamine; PC plas, phosphatidylcholine plasmalogen; PE plas, phosphatidylethanolamine plasmalogen; LECITHIN, PC + PC plas; CHOLINE, PC + PC plas + SPH (sphingomyeline); LECITHIN/CEPHALIN, (PC + PC plas)/(PE + PE plas); OUTSIDE, PC + SPH; INSIDE, PE + PS (phosphatidylserine); LEAFLET, (PC + SPH)/(PE + PS); ANIONIC/NEUTRAL, (PI (phosphatidylinositol) + PS + CL (cardiolipin) + PA (phosphatidic acid) + PG (phosphatidylglycerol))/(PC + PC plas + SPH + PE + PE plas); LYSO, LPC (lysophosphatidylcholine) + LPE (lysophosphatidylethanolamine). [b]$n = 12$. [c]$n = 25$. [d]$n = 18$. [e]F probability of analysis of variance. [f]$P<0.01$. [g]$P<0.05$. [h]Not detected in noninvolved tissues.

as a quantitative tool.

$^{31}$P MR phospholipid analysis permits the characterisation of the malignant, benign and noninvolved tissue types to the point that patterns in tissue spectra distinguish malignant from benign, malignant from noninvolved and benign from noninvolved (Figure 1). Observations made of differences in the order of relative intensities of phospholipids in the three tissue groups implies that differences exist among these tissue groups and that metabolism of phospholipids in the cell membrane of malignant tissue is aberrant. It should be noted that the aberrations in the patterns of signal intensity described in the results hold true for the computed mean relative concentrations.

*Fibrocystic disease*

Given the definition of fibrocystic disease as a premalignant process by the American Academy of Pathologists (1986), the finding that LPC and U independently predicted the classifications of fibrocystic disease and fibroadenoma in 92% of the 25 benign cases may indicate that these resonances in combination are a spectral sign of premalignancy. The uncharacterised resonance at 0.13 $\delta$ has no statistical significance in simple comparison of means; however, it is not only independently significant in differentiating fibrocystic disease from fibroadenoma, it is significant in differentiating benign from combined malignant and noninvolved tissue and noninvolved from neoplastic tissue in complex contrasts. This phospholipid awaits isolation and further characterisation.

*Phospholipid indices and complex contrasts*

Phospholipid indices calculated in this study and presented in Table IV are designed to facilitate the interpretation of the spectral data presented in Table I in terms of biochemical pathways or interrelationships. The indices are practical for analysing different aspects of phospholipid metabolism. The plasmalogens, for example, were only considered in those indices where their relationship with a regular phospholipid was established; therefore, the indices PC plas/PC and PE plas/PE are measures of the relationship of the more reduced enol-ether-containing plasmalogens to their more oxidised ester containing analogues in order to reflect the relative contribution of the metabolic pathways responsible for the biosynthesis of the ester-containing phosphatides and their corresponding enol ethers.

The use of complex contrasts in this analysis and in the general interpretation of MR spectral data is effective in that complex contrasts can define similarities and differences in processes or disease states of tissues. Not all of these differences and similarities can be explained in terms of known metabolic pathways or known alterations in membrane metabolism with disease. Indices are sometimes used to affirm or demonstrate trends in data. It should be noted that for the data presented in this study, when two complex contrasts are significant for a particular individual metabolite or index, the value of the noncombined group is less in one case and greater in the other. An example is PE, in which the noninvolved tissue level is significantly diminished compared to the neoplastic and the malignant is significantly elevated compared to the nonmalignant. The fact that a contrast cannot be interpreted in terms of a particular process should not detract from the value of contrast since it may represent processes unique to malignant or benign transformation.

*Choline-containing phosphatides*

Lysophosphatidylcholine, a product of phosphatidylcholine metabolism, is significantly decreased in both benign and malignant breast tissue. This is reflected in the complex contrast of normal and neoplastic tissues where the relative concentration of LPC in neoplastic tissues is depressed. Yamamoto and Ngwenya (1987) have reported that cancerous tissues release acyl-lysophospholipids as degradation products of acyl-phospholipids, such as those containing choline. The reduction in the relative levels of LPC in the neoplastic tissues suggests that there is an increase in the turnover of this acyl-lysophospholipid compared to noninvolved tissues.

Phosphatidylcholine plasmalogen (an alkyl-phospholipid), was significantly elevated in the malignant tissues compared to benign. Snyder and Wood (1968) demonstrated that malignant tissues release alkyl-phospholipids as degradation products of cell membranes. The complex contrast showing malignant tissue to be elevated *vs* nonmalignant at this resonance was significant which further distinguishes the phenomenon as an effect of malignancy.

The two major choline containing phospholipids, PC and SPH, studied as the index, OUTSIDE, represent the cell membrane's major outer leaflet phospholipid components. The identity of PC and SPH as outer leaflet components in malignant tissues (Widnell *et al.*, 1968) and other mammalian cells (Rothman *et al.*, 1977), and their position in the MR spectrum (Meneses *et al.*, 1988) are well established. This index is diminished in the malignant tissue compared with the benign, which implies that the choline incorporated into the phospholipids is inhibited in the malignant tissue or that the choline is being utilised in other processes. This finding is further confirmed in the complex contrasts of this index which shows the index to be significantly diminished in malignant tissue compared to nonmalignant and to be significantly elevated in the benign tissue compared to the combined malignant and noninvolved. This alteration in membrane asymmetry, specifically the components of the outer leaflet, may be a result of the production of degradation or substitution products, i.e. lysophosphatidylcholine and PC plasmalogen.

*Ethanolamine-containing phosphatides*

The relative concentration of phosphatidylethanolamine is significantly enhanced in malignant tissue compared to benign and noninvolved. This elevation is confirmed in the complex contrast of malignant tissue against nonmalignant tissue which adds to a previous finding (Merchant *et al.*, 1988) where the chemical residues of phosphatidylethanolamine, phosphorylethanolamine, glycerol 3-phosphorylethanolamine and α-glycerolphosphate were significantly elevated in malignant human breast tissues compared with benign and noninvolved tissues. This constellation implies that all the metabolites in phosphatidylethanolamine metabolism are elevated and is confirmed by the important role that PE has been found to play in the modification of membrane shape in malignant tissues (Cullis *et al.*, 1985). The presence of trace amounts of LPE in neoplastic tissues substantiates this conclusion.

The elevation of phosphatidylethanolamine and the reduction in choline-containing phosphatides SPH and PC, responsible for the bulk of membrane asymmetry in human tissues (Rothman *et al.*, 1977), provides a basis for study of changes in membrane fluidity and noncompensatory changes in membrane phospholipid composition with disease.

*Other phosphatides*

Phosphatidylinositol was significantly elevated in malignant tissues compared with benign, but it did not show significant difference with the normal tissue in simple comparison as a consequence of the conservative statistical methods used. PI, however, can differentiate malignant tissue from nonmalignant and benign tissue from combined malignant and normal. The simple observation in the spectra that a difference is present in the relative spectral PI signal intensity can be attributed to the activation of protein kinase C pathway in malignant tissues since PI is one of the activators of this pathway (Turner *et al.*, 1985; Price *et al.*, 1989).

We conclude that qualitative and quantitative analysis of the $^{31}$P MR phospholipid profiles of human breast tissues utilising the described techniques is a worthwhile addition to

MR spectroscopic analysis of diseased human breast tissues. The value of MR spectral data has been demonstrated by predicting the benign tissue histology in 92% of cases.

This research was supported by the intramural resources of the Pathology Institute, University of Utrecht and the Chicago College of Osteopathic Medicine.

### References

BARANY, M. & GLONEK, T. (1982). Phosphorus-31 nuclear magnetic resonance of contractile systems. In *Methods in Enzymology*, Frederiksen, D.L. & Cunningham, L.W. (eds). Part B, Vol 85, pp. 624–676. Academic Press: New York, NY.

BURT, C.T., GLONEK, T. & BARANY, M. (1976). Analysis of phosphatic metabolites, the intracellular pH, and the state of adenosine triphosphate in intact muscle by phosphorus nuclear magnetic resonance spectroscopy. *J. Biol. Chem.*, **251**, 2584.

COMAN, D.R. (1944). Decreased mutual adhesiveness: a property of cells from squamous cell carcinomas. *Cancer Res.*, **4**, 625.

CONSENSUS MEETING OF THE CANCER COMMITTEE OF THE COLLEGE OF AMERICAN PATHOLOGISTS (1986). Is fibrocystic disease of the breast precancerous. *Arch. Pathol. Lab. Med.*, **110**, 171.

CULLIS, P.R., HOPE, M.J., DE KRUIJFF, B., VERKLEIJ, A.J. & TILCOCK, C.P.S. (1985). Structural properties and functional roles of phospholipids in biological membranes. In *Phospholipids and Cellular Regulation*, Kuo, J.F. (ed.) pp. 1–59. CRC Press: Boca Raton, Florida.

DALY, P.F., LYON, R.C., FAUSTINO, P.J. & COHEN, J.S. (1987). Phospholipid metabolism in cancer cells monitored by $^{31}$P NMR spectroscopy. *J. Biol. Chem.*, **262**, 14875.

FOLCH, J., LEES, M. & SLOAN-STANLEY, G.H. (1957). A simple method for the isolation and purification of total lipids from animal tissues. *J. Biol. Chem.*, **226**, 497.

FREDDO, L., HAYS, A.P., NICKERSON, K.G. & 10 others (1986). Monoclonal anti-DNA IgMk in neuropathy binds to myelin and to a conformational epitope formed by phosphatidic acid and gangliosides. *J. Immunol.*, **137**, 3821.

GLONEK, T. & VAN WAZER, J.R. (1974). Aqueous tetrahydrophosphonium perchlorate as a narrow-line $^{31}$P NMR reference substance. *J. Magn. Reson.*, **13**, 390.

GREIG, R.G. & POST, E.G. (1986). Biological membranes and maligancy: an overview of pharmacological opportunities. In *Membrane Pathology*, Bianchi, G., Carafoli, E. & Scarpa, A. (eds). *Ann. NY Acad. Sci.*, **488**, 430.

GREINER, J.V., KOPP, S.J., SANDERS, D.R. & GLONEK, T. (1981). Organophosphates of the crystalline lens: a nuclear magnetic resonance spectroscopic study. *Invest. Ophthalmol. Vis. Sci.*, **21**, 700.

GUIDONI, L., MARIUTTI, G., RAMPELLI, G.M., ROSI, A. & VITI, V. (1987). Mobile phospholipid signals in NMR spectra of cultured human adenocarcinoma cells. *Mag. Res. Med.*, **5**, 578.

HEFTA, S.A., HEFTA, L.J.F., LEE, T.D., PAXTON, R.J. & SHIVELY, J.E. (1988). Carcinoembryonic antigen is anchored to membranes by covalent attachment to a glycosylphosphatidyl-inositol moiety: identification of the ethanolamine linkage site. *Proc. Natl Acad. Sci. USA*, **85**, 4648.

LOW, M.G. & SALTIEL, A.R. (1988). Structural and functional roles of glycosyl-phosphatidylinositol in membranes. *Science*, **239**, 268.

MENESES, P. & GLONEK, T. (1988). High resolution $^{31}$P NMR of extracted phospholipids. *J. Lipid, Res.*, **29**, 679.

MENESES, P., PARA, P. & GLONEK, T. (1989). $^{31}$P NMR of tissue phospholipids: a comparison of three tissue pre-treatment procedures. *J. Lipid Res.*, **30**, 458.

MERCHANT, T.E., GIERKE, L.W., MENESES, P. & GLONEK, T. (1988). P-31 MR spectroscopic profiles of neoplastic human breast tissues. *Cancer Res.*, **48**, 5112.

PATTON, S. & JENSEN, R. (1975). Lipid metabolism and membrane functions of the mammary gland. *Prog. Chem. Fats Lipids*, **14**, 1.

PRICE, B.D., MORRIS, J.D.H., MARSHALL, C.J. & HALL, A. (1989). Stimulation of phosphatidylcholine hydrolysis, diacylglycerol release, and arachidonic acid production by oncogenic ras is a consequence of protein kinase c activation. *J. Biol. Chem.*, **264**, 16638.

RONEN, S.M. & DEGANI, H. (1988). NMR of t47D human breast cancer cells grown as multicelluar spheroids. In *The Proceedings of the Society of Magnetic Resonance in Medicine*. p. 97.

ROTHMAN, J.E. & LENARD, J. (1977). Membrane asymmetry. *Science*, **195**, 743.

ROZENGURT, E. (1979). Early events in growth stimulation. In *Surfaces of Normal and Malignant Cells*, Hynes, R.O. (ed.), pp. 323–354. J. Wiley & Sons: New York, New York.

SIJENS, P.E., WIJRDEMAN, H.K., MOERLAND, M.A., BAKKER, C.J.G., VERMEULEN, J.W.A.H. & LUYTEN, P.R. (1988). Human breast cancer *in vivo*: H-1 and P-31 MR spectroscopy at 1.5 T. *Radiology*, **169**, 615.

SNYDER, F. & WOOD, R. (1968). The occurrence and metabolism of alkyl and alk-1-enyl ethers of glycerol in transplantable rat and mouse tumors. *Cancer Res.*, **28**, 972.

SPSS-X USER'S GUIDE (1986). Edition 2. McGraw-Hill Publishers New York.

TURNER, R.S. & KUO, J.F. (1985). Phospholipid-sensitive $Ca^{2+}$-dependent protein kinase (Protein kinase C): the enzyme, substrates and regulation. In *Phospholipids and Cellular Regulation*. Kuo, J.F. (ed.), Vol. II, p. 91. CRC Press: Boca Raton, Florida.

WIDNELL, C.C. & UNKELESS, J.C. (1968). Partial purification of a lipoprotein with 5′-nucleotidase activity from membranes of rat liver cells. *Proc. Natl Acad. Sci. USA*, **61**, 1050.

YAMAMOTO, N. & NGWENYA, B.Z. (1987). Activation of macrophages by lysophospholipids and ether derivatives of neutral lipids and phospholipids. *Cancer Res.*, **47**, 2008.